# The Effectiveness of Mindfulness-Based Interventions on Anxiety Disorders. A Systematic Meta-Review

**DOI:** 10.3390/ejihpe10030052

**Published:** 2020-07-14

**Authors:** Ascensión Fumero, Wenceslao Peñate, Cristián Oyanadel, Bárbara Porter

**Affiliations:** 1 Departamento de Psicología Clínica, Psicobiología y Metodología, Facultad de Psicología, Campus de Guajara, Universidad de La Laguna, 38200 Santa Cruz de Tenerife, Spain; wpenate@ull.es; 2 Facultad de Ciencias Sociales, Universidad de Concepción, 4030000 Concepción, Chile; coyanadel@udec.cl (C.O.); barbaraporter@udec.cl (B.P.)

**Keywords:** mindfulness, systematic review, meta-analysis, anxiety, effectiveness

## Abstract

Objective: There has been a growing interest in the study of the effectiveness of mindfulness-based interventions (MBIs). Many clinical trials and experimental designs have been implemented, with different samples and diverse MBI procedures. Reviews have shown unclear results, apart from a tendency to identify low-to-moderate effectiveness. The purpose of this review is to examine the effectiveness of MBIs on anxiety complaints, analyzing available systematic reviews and meta-analyses. Method: The literature search was done in MEDLINE (PubMed) and PsycINFO, from the first available review in 2003 until March 2020. From 82 initial references, 12 reviews were selected. Results: Reviews confirmed a moderate effect size of MBIs in improving anxiety symptoms. This efficacy was similar to that of well-established therapies for reducing anxiety symptoms, such as cognitive behavioral therapies. A large effect size was found when well-developed MBI protocols were applied. Discussion: More refined clinical trials are needed to establish clear conditions of MBI effectiveness (protocols, samples, psychological mechanisms, etc.). In addition, considering mindfulness processes, new outcome measures are needed (such as acceptance, self-awareness, or well-being) to test the incremental value of MBIs.

## 1. Introduction

The use of meditation as a therapeutic resource for physical and psychological problems has enjoyed appreciable growth in recent decades. Mindfulness represents a well-known meditation procedure in clinical practice. As is known, mindfulness is mainly based on Buddhist mind-body considerations. According to [1], this includes viewing human suffering as part of how people deal with some (negative) processes of the mind: human beings increase their suffering and distress when they focus on negative emotions. Mindfulness tries to teach a new way of relating with negative feelings through different processes and strategies (observing, describing, acting with awareness, nonjudging, and nonreacting) based on focusing on the present moment [2].

While the precise “active principle” behind mindfulness efficacy remains unclear, processes such as self-awareness, focused attention, and emotion regulation are frequently cited as playing a central role in the functioning of mindfulness [3]. In addition, acceptance processes are frequently included as the prevalent emotion regulation strategy in mindfulness meditation, as part of a wide range of mindfulness acceptance-based therapies [4]. Specifically, patients with anxiety disorders frequently focus on their anxiety symptoms as being representative of their suffering. Mindfulness practice teaches patients “to attend to a wide range of changing objects of attention while maintaining moment-to-moment awareness (mindfulness), rather than restricting one’s focus to a single object such as a mantra” (p. 937, [5]).

Although there are several forms for applying mindfulness (e.g., [6]), Mindfulness-based interventions (MBIs), mindfulness-based programs, mindfulness-based therapies, and mindfulness-based training are terms used to represent the strategies for applying this therapeutic resource. These include several procedures, such as mindfulness-based stress reduction (MBSR, [7]), mindfulness-based cognitive therapy (MBCT, [8]), mindfulness and acceptance-based intervention (MABI, [9]), and mindful self-compassion (MSC, [10]). It is likely that these protocols have favored the clinical application of mindfulness and studies of its effectiveness [2]. Therefore, several systematic reviews and meta-analyses have been carried out.

In general, MBIs have proven to be an effective therapeutic procedure for a variety of psychological and physical problems, usually with moderate effect sizes, including negative emotional strategies, such as rumination (e.g., [11]). In addition to being used for anxiety disorders, MBIs have also been applied to many health problems and disorders, such as depression, social functioning, prosocial behavior, pain, sleep disturbances, and problems in cancer sufferers [12].

Yet MBI applications have not always been considered effective, nor equally effective. For example, in Strauss, Cavanagh, Oliver, and Pettman’s review [13] there were significant effects of applying MBI on anxiety symptom reduction, but these were not sufficient when patients had a diagnosis of anxiety disorders; in Piet and Hougaard’s review [14], similar results were found for depressive symptoms; also, in Veehof, Oskam, Schreurs, and Bohlmeijer’s review [15], no significant effects were found in quality of life in patients with chronic pain. In Roche, Kroska, and Denburg’s review [16], there were not significant effects for smoking cessation (and a significant but slight effect for weight loss). In Kreplin, Farias, and Brazil’s review [17], no effects were observed of meditation on some prosocial behaviors such as aggression or prejudice. In addition, when some significant effects were found (on compassion and empathy), this was only observed when MBI effects were contrasted against a passive control group. Interestingly, these authors also found a controversial bias: when meditation teachers/trainers participated as co-authors of publication, more significant positive effects were found. On the other hand, no special adverse effects were observed as a consequence of meditation practice and, when these unwanted effects (such as anxiety/stress, depersonalization, or loss of consciousness/dizziness) were found, practitioners stated that these symptoms were transitory [18].

These disparities about the efficacy of MBIs can be ascribed to several reasons, most of which are related to conceptual clarity and methodological refinement. These include the use of different sample types: patients, subclinical samples, or non-clinical samples (e.g., [19]); differences in patient age (e.g., [20]); or the study of different meditation practices, with varying numbers of sessions, the absence of a comparison active control group, and no double-blind design [21]. Notably, the samples used in clinical trials or experimental research were diverse in their diagnoses; in addition, there were differences in diagnosis stage, with both acute and recurrent disorders covered (e.g., [22]).

These differences in the conditions led to different conclusions being drawn. This is especially relevant for anxiety disorders: in literature reviews focusing on different mental disorder studies, the largest effect size for MBIs was found for anxiety disorders, compared with other mental disorders (i.e., [23]), moderate effect size, comparable efficacy of other active treatments (i.e., [24]), and inconsistent results. Even a general moderate effect was found (i.e., [21,25]).

Given these disparities in the literature, the purpose of this review is to carry out a meta-review on the effectiveness of different MBI applications for anxiety disorders. It includes literature reviews directly related with MBIs and different anxiety disorders as well as general mental health reviews that provide specific and separate quantitative data for anxiety disorders.

## 2. Materials and Methods

### 2.1. Sources/Literature Research

A systematic literature search was performed in the MEDLINE (PubMed) and PsycINFO electronic databases. MEDLINE/PubMed is the premier bibliographic database dedicated to health studies in the behavioral sciences. Furthermore, this research included the main database in the field of psychology (PsycINFO).

Studies reported in English or Spanish were included, from the year for the first available review (2003), to March 2020. We did not predefine any dates for the search. The search strategy was developed for each electronic database using the combination of the following medical subject heading (MeSH) and free-text terms: anxiety disorders diagnostic ([anxi*] OR [Anxiety disorders] OR [phobi*] OR [Generalized Anxiety Disorder] OR [GAD] OR [panic] OR [compulsi* Disorder] OR [CD] OR [social phobia] OR [post-trauma*] OR [PTSD]) AND mindfulness intervention ([mindfulness-based strategies] OR [mindfulness-based treatments] OR [mindfulness-based interventions] OR [MBI] OR [mindfulness-based therapies] OR [mindfulness-based approaches] OR [mindfulness-based program] OR [mindfulness-based stress reduction] OR [MBSR] OR [mindfulness-based cognitive therapy] OR [MBCT]) AND meta-analysis ([meta-analysis] OR [MA] OR [meta-analytic study] OR [systematic review] OR [RS] OR [review]).

The search strategies were developed and tested. The references of selected articles were inspected. Initially, duplicates were removed from the total of identified records. Title and abstracts from the remaining records were then screened. Later, for assessment of eligibility the full-text articles were retrieved. Finally, studies fulfilling inclusion criteria were selected. The four authors verified the retrieval process.

### 2.2. Selection Criteria

Inclusion criteria: (i) Narrative review, scope studies, systematic reviews (SR), and meta-analytic studies (MA) examining the pre-post or controlled effects of MBIs for a wide range of psychological conditions related to anxiety disorders. (ii) Reviews published in peer-reviewed journals. (iii) Reviews examining nonrandomized and randomized controlled trials, experimental studies, and randomized clinical trials. (iv) Participants with a diagnosis of anxiety disorders or subclinical samples with higher levels of anxiety that can be signs of anxiety disorder. (v) Different types of intervention: mindfulness meditation, mindfulness-based cognitive therapy (MBCT), mindfulness-based stress reduction (MBSR), other types of MBIs, and mindfulness-based psychotherapy (MBP) interventions. (vi) Reviews selecting studies with comparison conditions: no intervention, control, waiting list, treatment as usual (TAU), other treatment group, or other active control groups. (vii) Studies including at least some of the following outcome measures: improvement in clinical anxiety scales, global anxiety improvement, anxiety improvement determined by clinician, and improvement in anxiety level specified by trials.

### 2.3. Exclusion Criteria

Studies were excluded if they (i) did not include at least one mindfulness-based intervention group; (ii) did not aim to examine treatment effects or reported no clinical outcomes or no measures of MBI anxiety effectiveness; (iii) were reviews about observational studies, cross-sectional studies or case-control designs; (iv) were comparisons among meditators or among meditation styles; (v) used non-mindfulness forms of meditation, such as transcendental meditation; or (vi) were reviews examining mindfulness as a component of another treatment; and (vii) were published in languages other than English or Spanish.

### 2.4. Study Selection and Data Extraction

All the authors assessed the eligibility criteria. The inter-rater reliability was not calculated but disagreements in the studies inclusion were resolved by consensus. Information was extracted from each included review that met the eligibility criteria, based on the following: name of the first author; year of publication; quantity and characteristics of the study (design, randomization); target population; implemented intervention; comparison group/s; effect size (Hedges’ g), 95% confidence interval, and *p*-values for each included study; main results after intervention and, if possible, follow-up.

### 2.5. Data Analysis and Synthesis

The data from the included reviews are presented descriptively following the structure of Table 1.

The reviews included showed whether a factor was described as having a positive or a negative influence on the implementation of intervention results. Due to the large variety of factors described and methods used, no quantitative pooling was performed across the reviews. Moreover, the large majority of the reviews studied did provide numbers, for example, in the form of effect sizes. Conclusions for the meta-review were therefore based on the conclusions and results presented in the reviews. The changes from baseline to post-intervention in anxiety and other continuous outcome measures were compared between participants who received MBI and those who received control/other interventions.

## 3. Results

We used Preferred Reporting Items for Systematic Reviews and Meta-Analyses (PRISMA) guidelines [37] to examine reporting in a systematic way.

**Figure 1:**
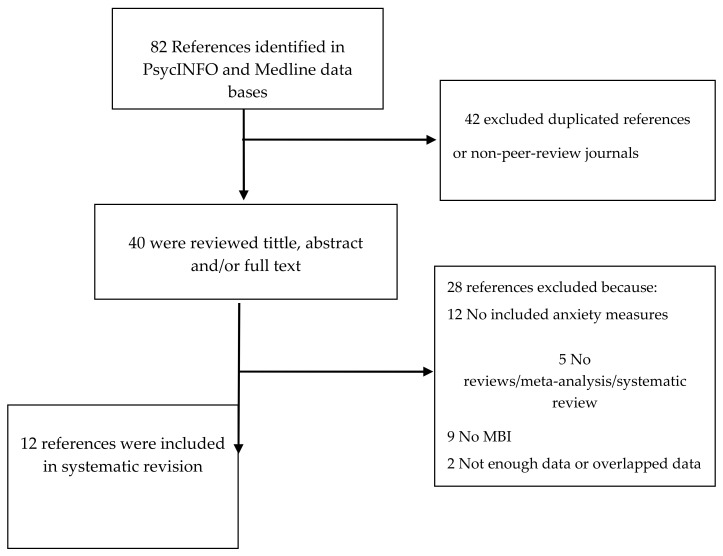
Study flow chart.

A total of 82 potentially relevant records were retrieved in the literature search. After screening all by title and abstract and removing duplicates and studies not subject to peer review, a total of 40 references were identified (Figure 1). Of these, most were excluded either because they did not report pertinent outcome measures, they did not refer to anxiety disorders, interventions based on the mindfulness technique were not applied, or not enough results data were provided. Twelve articles were considered eligible for inclusion through this combined search strategy. The methodological quality of these reviews was examined using the AMSTAR tool [38]. Table 2 summarizes the responses to the AMSTAR items. As can be observed, 10 reviews reached the required methodological quality in more than half of the items, and six of those studies obtained a positive rating in eight or more items. Only two reviews showed clear methodological insufficiencies.

In general, the reviews formulated a clear research question, searched for studies in appropriate electronic data sources, presented a comprehensive summary table (with the main data of studies selected), and, when applicable, used suitable methods to combine results. The more critical aspect has to do with the absence of a list of excluded studies, followed by the practical exclusion of grey literature.

### 3.1. Descriptive Characteristics

As shown in Table 1 the 12 reviews (ordered by publication date) comprise a total of 196 studies. When considered by category of design, eight reviews and meta-analyses (RSs/Mas) restricted selection to randomized controlled trials. The rest included non-randomized trials or pre-post experimental designs. Few reviews offered data about the methodological quality of the studies selected. In those that did, the quality ranged from moderate to low [21,29]. In the best review, 40% of studies were considered to be of high quality [39]. Studies included mainly samples with anxiety disorders, but also included anxiety symptoms within a wide range of psychological conditions [23,33].

The type of meditation reviewed was especially diverse. More than half of the reviews included studies with comparable protocols MBIs (MBSR, MBCT). In addition, comparison groups varied. MBIs were frequently compared with an inactive control group or TAU group. When studies included active control groups, those groups usually did not receive a protocolled alternative treatment. Five RSs/MAs included studies where MBIs were compared with well-established cognitive behavioral therapy (CBT) treatments [21,23,30,33,35].

Finally, referring to outcome measures, self-reported levels of anxiety were usually included (via inventories and scales). Few RSs/MAs selected studies had other dependent measures, such as attrition rates, adherence to treatment, or well-being/quality of life [13,33]. The contrasts for those outcome measures were provided in Hedges’ g or Cohen’s d.

### 3.2. MBI Effectiveness

A total of 9 of the 12 (75%) reviews indicated a positive effect of MBIs, comparing pre-post intervention anxiety scores and compared with a control group. In three SRs/MAs, with MBI vs. comparison group, the effect size did not reach a significant level, or the results were equivalent between MBI group and comparison group. This equivalence was especially true when the comparison group included some CBT intervention [13,21,32,37].

For RSs/MAs with positive results for mindfulness, the weighted mean effect size was standardized mean difference (SMD) = 0.57 (95% CI = 0.22–0.89); for those with negative results, SMD = −0.27 (95% CI: −0.52–0.02). Participants receiving MBI improved their anxiety levels, with a medium effect size.

For the studies with positive results for mindfulness in comparison to control or other intervention groups, the range of effect size was large for 20% of studies [23,30], moderate for 50% [26,29,32,33,39], and small for 30% [31,34,36]. Non-favorable comparisons were found by Strauss, Cavanagh, Oliver, and Pettman [13], with non-significant post-MBI between-group differences in anxiety symptom severity; by Singh and Gorey [35], with no significant differences observed between the mindfulness and CBT groups on anxiety levels; and by Goldberg et al. [21], where mindfulness-based interventions were equivalent both to the comparison group and to evidence-based treatment. These different types of effectiveness are not associated with the methodological characteristics of these reviews; in each effectiveness category (positive, equal to other treatment, non-favorable), reviews with different methodological qualities are mixed.

Reviews considering samples with a clinical diagnosis of anxiety disorders showed a positive effect of MBI, but with several nuances. Baer [26], Hoffmann et al. [29] and Kishita et al. [33] obtained a moderate effect size. Galante et al. [31] found significant but unstable positive effects (depending on the studies selected). In Goldberg et al. [21], Strauss et al. [13], and Hodann-Caudevilla and Serrano-Pintado [32], MBI effectiveness was not superior to CBT intervention.

MBI significantly reduced anxiety levels, with moderate effect sizes. Reviews about the application of standard protocols (MBSR, MBCT) found more diverse effect sizes, and several RSs/MAs did not find significant differences between MBSR/MBCT and other active treatments in reducing anxiety levels [13,23].

Finally, the reviews verified the frequent absence of follow-up in the studies selected. When follow-up was included, the results pointed to the maintenance of moderate effectiveness of MBI [23], with moderate (median 15%) attrition rates [13].

## 4. Discussion

The therapeutic effectiveness of MBIs has been assessed through multiple clinical trials (randomized and non-randomized) and experimental designs. In general, data support its efficacy, frequently with a moderate effect size, but there still are inconclusive results. This is especially true when MBIs are used to improve anxiety levels. An appreciable number of systematic reviews and meta-analyses have been published to determine MBIs efficacy on anxiety problems. The outcomes of these reviews do not reach clear conclusions. In that sense, this paper has analyzed those literature reviews, trying to find a synthesis of the major results of MBIs on anxiety-related problems, and searching for conditions where MBIs show a better efficacy.

Twelve reviews were selected. In general, there is a clear tendency to consider MBIs as an effective resource for reducing anxiety problems; MBIs improve anxiety levels, as seen when comparing pre-post intervention scores or comparing mindfulness with an inactive control or TAU group. This effectiveness tends to reach moderate effect sizes. In addition, these effects—when data were available—persist in follow-up, with moderate drop-out rates. This finding was verified in several randomized controlled trials (i.e., [31,33,36]). Furthermore, when MBIs are compared with well-established therapies for anxiety disorders (especially CBTs), there are no differences observed between them (i.e., [35]). However, when MBIs are provided via the Internet, their efficacy is less positive [34]. Apparently, the methodological quality of these reviews is not associated with a specific MBI effectiveness level but with the absence of a list of excluded studies and the exclusion of grey literature.

Nevertheless, the literature reviews included in this review also show some inconsistencies. As Hoffmann et al. [29] point out, MBIs are useful for reducing anxiety levels when participants have a high level of anxiety, but MBIs do not have enough power when they are applied to individuals with a diagnosis of anxiety disorders. Another insufficiency is related with the effect size obtained by applying MBIs. Few studies found large effect sizes. Several CBTs obtained similar (or better) results. Therefore, according to incremental validity, what reasons make MBIs eligible? According to data provided by Khoury et al. [23], large effect sizes are obtained when standard protocols of mindfulness are applied (such as MBSR or MBCT). However, if we examine the kind of meditation used, there are still several experimental studies using different meditation procedures [39]; these procedures are frequently not comparable, nor are the duration, number, or content of sessions the same (e.g., transcendental meditation, yoga, or tai-chi). This represents a relevant methodological problem.

If we consider the type of participants, a generalized positive effect of MBIs is observed that is not related to the nature of the sample. Anxiety reduction can be detected in samples with medical or mental problems and in non-clinical samples [23]. Yet, as mentioned above, improvements are insufficient to generate a clinical change when participants have a diagnosis of anxiety disorder [29]. This last result can hide a methodological problem: frequently reviews include samples with anxiety disorders, as a general label (where different anxiety disorders were incorporated); but it is possible MBIs can differentially affect anxiety, depending on the type of anxiety disorder (panic, generalized, etc.). Unfortunately, with the available data provided from reviews, we cannot answer this hypothesis. Although, this data can represent an opportunity for an epistemological change to how MBIs are evaluated: the main outcome measure used to assess MBI effectiveness (and other therapies) is symptom reduction. This is obvious, because this measure is directly related with human suffering. Yet mindfulness meditation also implies other changes, according to its conceptual foundations. These changes are related to the use of acceptance as a strategy for emotion regulation, self-awareness, or well-being. In this sense, measuring these variables can be an opportunity to highlight the incremental value of applying MBIs. Secondary outcome measures, such as relapse prevention, adherence to treatment, or attrition rates [13,33] can participate in this incremental value.

Finally, little attention is paid to relevant variables, such as type of therapist, cost-effectiveness, or the reasons that make MBIs work. As Kreplin et al. [17] critically pointed out, the type of therapists and their training (as teachers, psychologists, or other) can play a relevant role in the effectiveness of MBIs. What is more, Carsley, Khoury, and Heath [40] found a differential effectiveness that was dependent on the type of trainer/therapist. Singh and Gorey [35] pointed out that MBIs are cost-effective, but that more precise studies are needed, with an objective methodology (and comparing MBIs with other effective therapies such as CBT). As mentioned in the introduction, there are several supposed active principles explaining why mindfulness works [3]. As Norton [41] states, there are enough data to hypothesize how some strategies can be more effective, but there are no trials testing different mindfulness strategies.

According to these data, we think future reviews could deal with more methodologically refined studies. When a bias analysis was done, the methodological quality was found to be medium or low. As Goldberg et al. [21] pointed out, despite the interest in improving MBI design, relevant methodological problems remain. In this sense, reviews of well-designed randomized clinical trials, with comparable MBI protocols (preferably MBSR or MBCT), active control groups (especially groups receiving CBTs), follow-up, and with clinical samples, can offer more relevant and clear results about the effectiveness of MBIs. Analysis of outcome measures could provide both statistical and clinical efficacy. Furthermore, analysis of outcome measures indirectly related with treatment efficacy (attrition rates, adherence) could modify MBI effectiveness criteria. Finally, studies about the “active principle” of mindfulness are sorely lacking, and if there are different mechanisms according to MBI protocol and type of treated problem. These mechanisms could also observe the role of mindfulness as a protective/preventive resource for mental and biomedical problems [42,43].

This meta-review has several limitations. The selected RSs/MAs were directly related to anxiety problems. Anxiety disorders can include different anxiety processes (panic disorders, generalized anxiety disorders, etc.), and it is possible to think MBIs can differentially affect those processes involved. Studies included in several reviews were with subclinical samples or included samples with different physical and mental complaints, and so it is possible that these interventions have differential effectiveness, depending on the target problem. Studies were obtained from peer-reviewed journals, but there may be many unpublished or online trials not collected in this review. Not all the studies extracted share a similar MBI procedure, and it can be questionable whether those procedures were really comparable. In spite of the number of search literature databases established, the present search was done in two major databases; thus, the results could be constrained because of the remaining databases excluded.

## 5. Conclusions

Several general conclusions can be drawn: (i) The RSs and MAs reviewed confirm the existence of well-designed randomized controlled trials (RCTs) testing the effectiveness of different MBIs. (ii) These reviews point out the significant efficacy of MBIs in improving anxiety symptoms. However, this efficacy is similar to that obtained with traditional CBT. (iii) Large effectiveness was obtained when well-developed MBI protocols were applied, and with non-clinical anxiety samples. (iv) Existing data support high adherence to MBIs. Finally, (v) incremental gains of MBI application could be found in variables closer to mindfulness processes, such as self-awareness, acceptance, and well-being.

## Figures and Tables

**Table 1:** Summary of studies included in the review.

First Author and Year	Studies Included	Target Population	Implemented Intervention	Comparison Group	Effect Size	Main Results
[26]	21 uncontrolled and controlled studies, but only two were based on anxiety disorder	Anxiety clinical sample	MBSR	None	d = 0.70	Kabat-Zinn et al. [27] examined patients with generalized anxiety and panic disorders and found significant improvements (also at 3-month follow-up). Miller, Fletcher, and Kabat-Zinn [28] reported a 3-year follow-up of the same participants and results were maintained.
[29]	39 uncontrolled and controlled studies	Anxiety clinical sample	MBSR or MBCT	None, TAU, educational social support with relaxation, anxiety education program, waiting list	For anxiety disorders, ES estimates suggest that mindfulness-based therapy was moderately effective for improving anxiety (Hedges’ g = 0.63; 95% CI = 0.47 to 0.87) from pre- to post-treatment in the overall sample	The uncontrolled pre-post ES estimates were in the moderate range for reducing anxiety symptoms. MBT in patients with anxiety disorders was associated with a large ES.
[30]	19 controlled and uncontrolled trials	Individuals with clinical levels of anxiety	Multi-component acceptance-based interventions (CBT)	Stand-alone mindfulness	Between groups Hedges’ g = −0.83; 95% CI = −1.62 to −0.04	MBIs are associated with robust and substantial reductions in symptoms of anxiety. No significant differences emerged between stand-alone mindfulness interventions and multi-component treatment packages.
[31]	11 randomized controlled trials	Patients diagnosed with anxiety disorders	MBCT	TAU	The average degree of anxiety decreased compared to TAU (Hedges’ g = −0.42; 95% CI = −0.74 to −0.09)	Anxiety obtained significant but unstable results in sensitivity analyses comparing additive MBCT against usual treatment.
[23]	209 waiting list-controlled studies but only 32 focused on anxiety	Medical conditions and non-clinical population with elevated initial anxiety	MBI	Pre-post studies, waiting list controlled and psycho-educational interventions, supportive therapies, relaxation and imagery/suppression technique	The SMD was large (10 studies pre-post) for anxiety studies (Hedges’ g = 0.89; 95% CI = 0.71 to 1.08) and for 4 waitlist-controlled studies (Hedges’ g = 0.96 (95% CI = 0.67 to 1.24)	MBT is moderately effective in pre-post comparisons, in comparisons with waitlist controls and when compared with other active treatments, including other psychological treatments. MBT did not differ from traditional CBT or behavioral therapies or pharmacological treatment. MBT was associated with the largest mean ES for anxiety.
[13]	12 randomized controlled trials; 9 included a measure of anxiety symptoms	Full diagnostic criteria for anxiety	MBCT, MBSR and person-based cognitive therapy	Active control conditions (psychoeducation) and inactive control conditions (waiting list, aerobic exercise)	There was a non-significant post-MBI between-group difference in anxiety symptom severity (Hedges’ g = −0.52; 95% CI = −1.11 to 0.06). MBCT vs. inactive control (Hedges’ g = −1.03; 95% CI = −0.40 to −1.66). MBCT vs. active control (Hedges’ g = 0.03; 95% CI = 0.54 to −0.48).	There were no significant post-intervention between-group benefits of MBIs relative to inactive control conditions on anxiety symptom severity nor was there was an active control.
[32]	8 randomized and non-randomized clinical trials	Anxiety clinical sample	ACT, MBCT, MBSR	Waiting list, TAU, psychoeducation, CBT, aerobic exercises, relaxation	It was suggested that psychological interventions based on mindfulness constitute an effective treatment for GAD (from d = 0.92 to d = 3.4), SP (from d = 0.41 to d = 0.78), and PTSD (d = 0.63) when used as adjuncts to pharmacological treatment	The interventions based on mindfulness constitute an effective treatment for GAD, SP, and PTSD, when used as adjuncts to pharmacological treatment. However, an ES that combines the significant differences obtained for each of the disorders is not provided. For the comparison between treatments based on mindfulness and other treatments for anxiety (CBT, applied relaxation, and aerobic exercise), it is suggested that the former is not superior to the latter in terms of efficacy. Both MBSR and MBCT seem highly efficient interventions.
[33]	7 randomized controlled trials (RCTs)	Anxiety symptoms with a wide range of physical and psychological conditions	MCBT and ACT	Pre-post studies, control and active control groups	ES varied from not effective (Hedges’ g = 0.23) to large and positive (Hedges’ g = 1.90). The random effect model showed an overall moderate ES (Hedges’ g = 0.58; 95% CI = 0.27 to 0.88) of mindfulness-based CBT for anxiety symptoms among older adults	Effect-size estimates suggest that mindfulness-based CBT is moderately effective on anxiety symptoms in older adults (g = 0.58)
[34]	15 RCTs, 11 comparisons on anxiety	Anxiety clinical sample	ACT, MBCT, MBSR, Internet-based Mindfulness treatment	Control group	Based on 11 comparisons, a significant, small ES was found for online MBIs on anxiety, with g = 0.22 (95% CI = 0.05 to 0.39, *p* = 0.010) and no outliers. After removal of low-quality studies from the analysis, the ES was virtually the same (g = 0.21, 95% CI = 0.03 to 0.40, *p* = 0.022).	A small but significant ES was found on anxiety. The online MBIs are not as effective as traditional face-to-face MBIs in reducing anxiety.
[21]	142 randomized clinical trials (18 based on anxiety disorders)	Anxiety clinical sample	MBI	No treatment, specific active control, evidence-based treatment	For anxiety, MBIs were equivalent to the comparison group (d = 0.15 (95% CI = −0.16 to 0.46) and were equivalent to EBTs (d = −0.18 (95% CI = −0.41 to 0.06)	Mindfulness-based interventions were equivalent to the comparison group and EBTs for anxiety
[35]	9 randomized trials	Anxiety clinical sample	MBI	CBT (active control groups)	Between groups Cohen’s d = −0.02; 95% CI = −0.16 to 0.12	No statistically or practically significant differences between mindfulness and cognitive behavioral intervention
[36]	10 randomized controlled trials	Anxiety clinical sample	MBCT and MBSR	Control conditions, CBT	MBIs were superior to control interventions for internalizing (SE = 0.26; 95% CI = 0.64 to 0.12; *p* = 0.00) and distress (SE = 0.12; 95% CI = 0.7 to 0.21; *p* = 0.00), but not for fear symptoms (SE = 0.22; 95% CI = 0.45 to 0.4; *p* = 0.90). A significant difference that favor CBT over MBIs for the fear domain symptoms were found (SE = 0.1; 95% CI = 0.1 to 0.46; *p* = 0.00). No evidence for superiority of CBT over MBIs was found.	MBIs were superior to control interventions for internalizing and distress, but not for fear symptoms. CBT was superior to MBIs for fear symptoms but not for internalizing and distress.

Notes: CBT = cognitive behavioral therapy; MBI = mindfulness-based interventions; MBCT = mindfulness-based cognitive therapy; MBSR = mindfulness-based stress reduction; CBGT = cognitive behavioral group therapy; SMD = standardized mean difference; TAU = treatment as usual; RCT = randomized controlled trial; CI = confidence interval; NA= not available; ES = effect size; ACT= acceptance and commitment therapy; GAD = generalized anxiety disorder; SP = social phobia; PTSD = posttraumatic stress disorder; EBT = Evidence-based treatment.

**Table 2:** Methodological quality of selected review studies, using the AMSTAR tool.

AMSTAR Items												
First Author and Year	1	2	3	4	5	6	7	8	9	10	11	Total YES
[26]	YES	NO	YES	NO	NO	YES	NO	NO A	YES	NO	C N A	4
[29]	YES	YES	YES	NO	NO	YES	YES	YES	YES	NO	YES	8
[30]	YES	YES	YES	NO	NO	YES	NO	NO A	YES	YES	C N A	6
[31]	YES	YES	YES	NO	NO	YES	NO A	NO A	YES	YES	YES	7
[23]	YES	YES	YES	NO	NO	YES	YES	YES	YES	YES	YES	9
[13]	YES	C N A	YES	YES	NO	YES	YES	C N A	YES	YES	C N A	7
[32]	YES	C N A	YES	NO	NO	YES	NO	NO A	N A	NO	YES	4
[33]	YES	YES	YES	YES	NO	YES	C N A	YES	YES	YES	YES	9
[34]	YES	YES	YES	NO	NO	YES	YES	YES	YES	YES	C N A	8
[21]	YES	YES	YES	YES	NO	YES	YES	YES	YES	NO	YES	9
[35]	YES	C N A	YES	NO	NO	YES	NO	N A	YES	YES	C N A	5
[36]	YES	YES	YES	NO	NO	YES	YES	YES	YES	YES	YES	9

Notes: AMSTAR items: 1. Was an “a priori” design provided?; 2. Was there duplicate study selection and data extraction?; 3. Was a comprehensive literature search performed?; 4. Was the status of publication (i.e., grey literature) used as an inclusion criterion?; 5. Was a list of studies (included and excluded) provided?; 6. Were the characteristics of the included studies provided?; 7. Was the scientific quality of the included studies assessed and documented?; 8. Was the scientific quality of the included studies used appropriately in formulating conclusions?; 9. Were the methods used to combine the findings of studies appropriate?; 10. Was the likelihood of publication bias assessed?; 11. Were potential conflicts of interest included? C N A = Cannot answer; N A = Not applicable.
